# Dihydrochalcones and Diterpenoids from *Pteris ensiformis* and Their Bioactivities

**DOI:** 10.3390/molecules22091413

**Published:** 2017-08-25

**Authors:** Yu-Sheng Shi, Yan Zhang, Wen-Zhong Hu, Xiu-Fu Zhang, Xin Fu, Xia Lv

**Affiliations:** 1 Key Laboratory of Biotechnology and Bioresources Utilization, Educational of Minister, College of Life Science, Dalian Nationalities University, Dalian 116600, China; shiyusheng@dlnu.edu.cn (Y.-S.S.); shiyusheng_imm@163.com (X.-F.Z.); lvxia@dlnu.edu.cn (X.L.); 2 State Key Laboratory of Bioactive Substance and Function of Natural Medicines, Institute of Materia Medica, Chinese Academy of Medical Sciences and Peking Union Medical College, Beijing 100050, China; 3 Jiamusi College, Heilongjiang University of Chinese Medicine, Jiamusi 154007, China; 4 Department of Pharmacognosy, Heilongjiang University of Chinese Medicine, Harbin 150040, China; fuxinhlj@sina.com

**Keywords:** *Pteris ensiformis*, dihydrochalcone, diterpenoid, inhibitory activity on nitric oxide production, cytotoxic activity

## Abstract

Two new dihydrochalcone enantiomers (+)-**1** and (−)-**1**, along with eight known compounds **3**–**10**, were obtained from *Pteris ensiformis*. The planar structures were determined on the basis of extensive 1D and 2DNMR and HRESIMS. The resolution of (+)-**1** and (−)-**1** was achieved by chiral HPLC analysis. The absolute configurations of (+)-**1** and (−)-**1** were established by the bulkiness rule using Rh_2_(O_2_CCF_3_)_4_-induced circular dichroism (ICD) method. Compounds (+)-**1**, (−)-**1**, **8**, **9** and **10** exhibited the inhibitory assay of NO production in mouse macrophages stimulated by LPS, with IC_50_ values of 2.0, 2.5, 8.0, 9.5 and 5.6 μM, respectively. Otherwise, compound **10** showed moderate cytotoxic activity against HCT-116, HepG-2 and BGC-823 cell lines with IC_50_ values of 3.0, 10.5 and 6.3 μM, respectively.

## 1. Introduction

*Pteris ensiformis* belongs to the family Pteridaceae, which is broadly distributed in the south of China, the north of India, and Malay Peninsula [1]. In folk medicine it has been used to cure dysentery, and has heat-clearing and detoxifying effect [2]. The whole plant is one of the most popular herbs used in beverages in Taiwan [3]. Previous phytochemical investigation of the genus *Pteris* have revealed numerous diterpenoids and flavonoids, some of which showed anti-inflammatory or anti-tumor activities [4,5,6,7,8]. In our previous study, a new *ent*-kaurane diterpenoid, *ent*-kaurane-6β,16α-diol-3-one, were isolated from the extract of *P. ensiformis* along with five known diterpenoids and three known sesquiterpenes [9]. Bioassays showed that 95% EtOH and polyamide resin at 30% ethanol extracts of *P. ensiformis* exhibited moderate anti-inflammatory activity against croton oil-induced ear edema in mice, with inhibition rates of 38.6% and 56.3%, respectively, at a dose of 200 mg/mL. To search for further bioactive constituents, a 95% EtOH extract of *P. ensiformis* was now investigated. As a result, two new dihydrochalcones (+)-**1** and (−)-**1**, along with eight known compounds **3**–**10**, were obtained from *P. ensiformis*. All the isolated compounds were evaluated for their inhibitory effects on macrophage activation by an inhibitory assay of nitric oxide (NO) production in mouse macrophages stimulated by lipopolysaccharide (LPS) and cytotoxic activity against three cancer cell lines. Thus, in the paper, we report the isolation, structure elucidation, inhibitory activity on nitric oxide production and antitumor activity of the isolated compounds.

## 2. Results and Discussion

**Figure 1:**
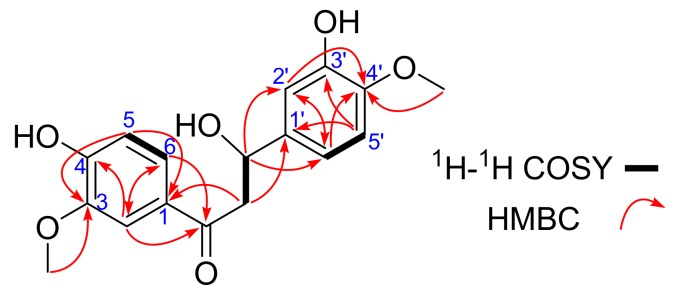
^1^H-^1^H COSY and HMBC correlations of **1**.

**Figure 2:**
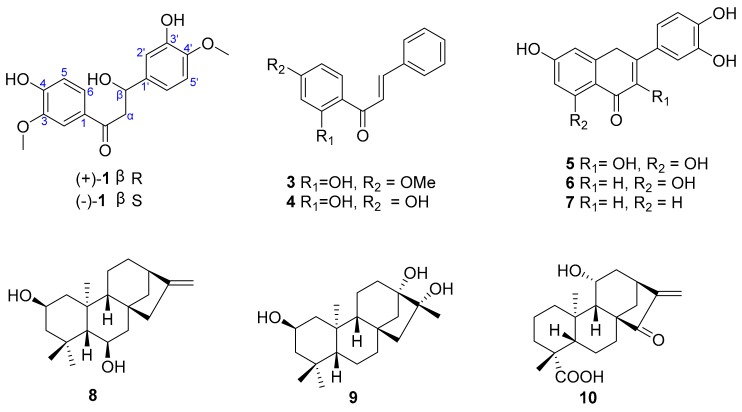
Structures of compounds **1**–**10**.

Compound **1** was obtained as white powder. Its molecular formula of C_17_H_18_O_6_ was established by ^13^C-NMR and HR-ESI-MS data (*m*/*z* 411.0721 [M − H]^−^, calcd. for 411.0722), indicating nine degrees of unsaturation. Its IR spectrum (see Table 1 and Figure S1) suggested the presence of hydroxy (3376 cm^−1^) and benzene ring (1660 cm^−1^) functionalities. ^1^H-NMR (see Table 1 and Figure S2) spectrum exhibited six aromatic hydrogens (*δ*_H_ 7.56 (1H, d, *J* = 2.0 Hz, H-2), 6.80 (1H, d, *J* = 8.5 Hz, H-5) and 7.62 (1H, dd, *J* = 8.5, 2.0 Hz, H-6)) and (*δ*_H_ 6.89 (1H, d, *J* = 2.0 Hz, H-2′), 6.73 (1H, d, *J* = 8.5 Hz, H-5′) and 6.76 (1H, dd, *J* = 8.5, 2.0 Hz, H-6′)), indicating existing two ABX spin systems, which was also partly supported by the correlation of H-5/H-6, and H-5′/H-6′ in the ^1^H-^1^H COSY spectrum (Figure 1). The correlations of H-α (*δ*_H_ 4.26 (1H, dd, *J* = 11.0, 8.5 Hz, H-α) and 3.71 (1H, dd, *J* = 11.0, 5.5 Hz, H-α)) and H-β (*δ*_H_ 4.76 (1H, dd, *J* = 8.5, 5.0 Hz, H-β)) suggested a linked -CH_2_(α)-CH(β)- moiety. In the HMBC spectrum, the correlation from H-β to C-2′ (*δ*_C_ 112.7) and C-6′ (*δ*_C_ 122.2), from H-α to C-1′ (*δ*_C_ 129.9) and C=O (*δ*_C_ 199.6), from H-6 and H-2 to C=O, from H-5 to C-3 (*δ*_C_ 149.0) and C-1 (*δ*_C_ 130.3), from H-5′ to C-3′ (*δ*_C_ 149.3) and C-1′ resulted to form the nuclear skeleton of compound **1**. Two cross peaks in the HMBC spectrum—OCH_3_ (*δ*_H_ 3.82)/C-4′, OCH_3_ (*δ*_H_ 3.86)/C-3—indicated the two methoxy groups were linked to C-4′ and C-3, respectively. Thus, the planar structure of compound **1** was established as shown in Figure 2.

**Figure 3:**
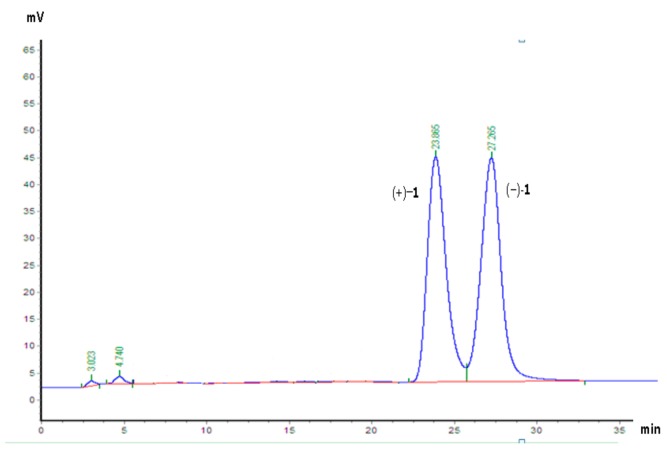
The HPLC separation chromatogram of (+)-**1** and (−)-**1** on chiral AD-H column (5 μm, 250 × 4.6 mm). Mobile phase, hexane/2-propanol = 4:1; flow rate: 1 mL/min; UV detection at 210 nm.

**Figure 4:**
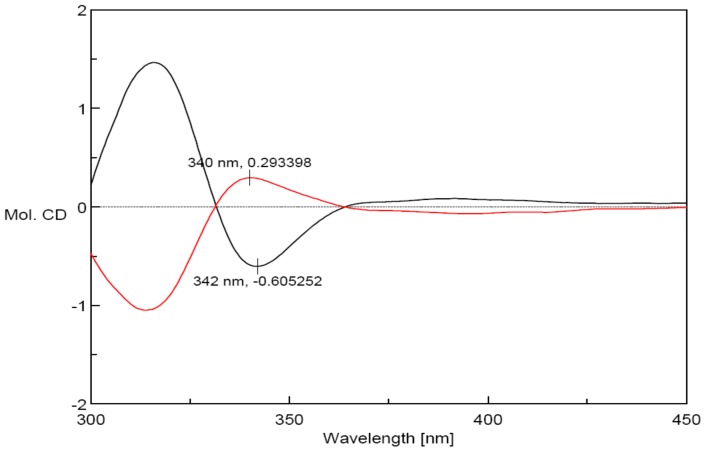
The Rh_2_(O_2_CCF_3_)_4_ Induced CD Spectra of (+)-**1** (black) and (−)-**1** (red).

The zero [α] suggested that compound **1** was a racemate. Sequent chiral analysis allowed the separation of the enantiomers, (+)-**1** ([α]25 *D* + 15.0 (*c* 0.2, CH_3_OH)) and (−)-**1** ([α]25 *D* − 15.1 (*c* 0.2, CH_3_OH)) (Figure 3). Owing to the fact compound **1** only possesses one chiral center (C-β), the absolute configurations of these enantiomers must be (β*R*)-**1** or (β*S*)-**1**. The absolute configuration of each enantiomer was determined by the Rh_2_(OCOCF_3_)_4_-induced circular dichroism spectroscopy technique. The results displayed negative and positive Cotton effects at 340 nm (Figure 4), respectively.

**Figure 5:**
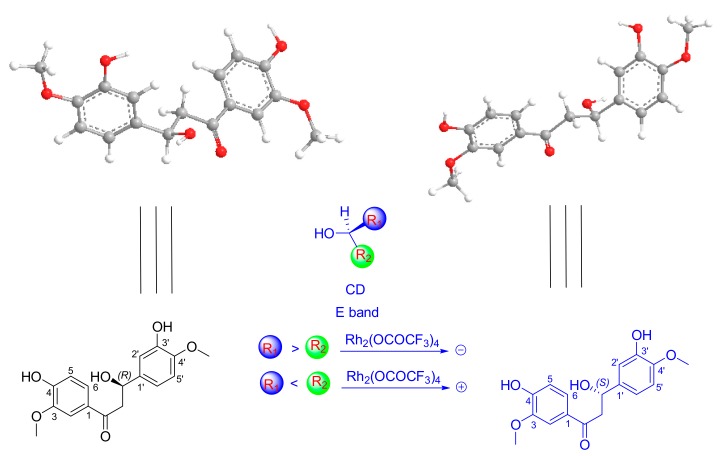
The conformations of (+)-**1** and (−)-**1**, and the application of the bulkiness rule for tertiary alcohols.

According to the bulkiness rule, the E band (near 350 nm) was demonstrated to be useful for determining the absolute configuration of chiral secondary and tertiary alcohols [10]. Thus, (+)-**1** and (−)-**1** were determined to be (+)-(β*R*)-3′,4-dihydroy-3,4′-dimethoxy-dihydrochalcone and (−)-(β*S*)-3′,4-dihydroy-3,4′-dimethoxy-dihydrochalcone by applying the bulkiness rule (Figure 5), respectively.

The eight known compounds: 2′-hydroxy-4′-methoxychalcone (**3**) [11], 2′,4′-dihydroxychalcone (**4**) [12], quercetin (**5**) [13], 5,7,3′,4′-tetrahydroxyflavone (**6**) [13], 7,3′,4′-trihydroxyflavone (**7**) [13], multikaurane B (**8**) [14], henrin A (**9**) [15], and *ent*-11α-hydroxy-15-oxokaur-16-en-19-oic acid (**10**) [16], were identified by comparing their spectroscopic data with literature data. Compounds (+)-**1** and (−)-**1** are dihydrochalcones, **3** and **4** are hydrochalcones, **5**–**7** are flavonoids, and **8**–**10** belong to the diterpenoid class.

In order to study the biological activity, results of an in vivo study showed that 95% EtOH and polyamide resin at 30% ethanol extracts of the plant exhibited anti-inflammatory activities against ear edema, with inhibition rates of 38.6% and 56.3%, respectively, at a dose of 200 mg/kg (Table 2).

Macrophages may be a potential therapeutic target for inflammatory diseases [17]. Activated macrophages release pro-inflammatory mediators, such as NO, reactive oxygen, interleukin-1 beta, tumor necrosis factor-alpha, and other inflammatory mediators, which play important roles in biological defense. However, the overexpression of these mediators had been implicated in diseases such as osteoarthritis, rheumatoid arthritis, and diabetes because the increased production of pro-inflammatory mediators has been shown to induce severe or chronic inflammation [17]. Isolated compounds **1**–**10** were evaluated for macrophage activation by the inhibitory assay of NO production in mouse macrophages stimulated by LPS. The result displayed that compounds (+)-**1**, (−)-**1**, **8**, **9** and **10** exhibited inhibitory effects on macrophage activation, with IC_50_ values of 2.0, 2.5, 8.0, 9.5 and 5.6 μM, respectively. However, others were inactive. Furthermore, compounds **1**–**10** did not show cytotoxic activity against mouse macrophage cells. The cell viability after treatment with each compound, even at a sample concentration of 125 μM, was more than 97%. Dexamethasone, with an IC_50_ value of 0.025 μM, was used as the positive control (Table 3).

All the compounds were next evaluated for their antitumor activity against three cancer cell lines, HCT-116, HepG2 and BGC-823. As a result, compound **10** exhibited moderated cytotoxic activity against HCT-116, HepG-2 and BGC-823 cell lines with IC_50_ values 3.0, 10.5 and 6.3 μM, respectively. Others were inactive (IC_50_ > 10 μM). Taxol, with IC_50_ values of 0.03, 0.012 and 0.004 μM, respectively, was used as the positive control.

## 3. Experimental Section

### 3.1. General Information

1D and 2D spectra were run on a Mercury-400 spectrometer (Varian, Palo Alto, CA, USA). Optical rotations were obtained on a P2000 automatic digital polarimeter (Jasco, Tokyo, Japan). IR spectra were obtained by a Thermo Nicolet 5700 FT-IR spectrometer using KBr pellets (Nicolet, Madison, WI, USA). HRESIMS was recorded on an Agilent 6520 Accurate-Mass Q-TOF LC/MS spectrometer (Agilent, Santa Clara, CA, USA). CD spectra were recorded on a JASCO J-815 spectropolarimeter (Jasco, Tokyo, Japan). Column chromatography (CC) was performed on polyamide resin (60–90 mesh, Zhejiang Taizhou Sijiashenghua Ltd., Taizhou, China), and MCI GEL CHP20P resin (75–150 μm, Mitsubishi, Tokyo, Japan). Fractions were monitored by TLC (GF 254, Qingdao Haiyang Chemical Co., Ltd. Qingdao, China), and spots were visualized by 10% H_2_SO_4_-ethanol reagent. Medium pressure liquid chromatography (MPLC) was employed using a Biotage pump system (Biotage, Uppsala, Sweden) coupled with C_18_-silica gel-packed glass column (YMC C-18, 500 × 50 mm, 50 μm, YMC, Tokyo, Japan). Preparative high performance liquid chromatography (Pre-HPLC) was performed using a Shimadazu LC-6A (SPD-10A) (Shimadazu, Tokyo, Japan), using YMC-Pack ODS-A (250 × 20 mm, 10 μm) and YMC C18 (250 × 20 mm, 5 μm) chromatographic column (YMC). An Agilent 1260 HPLC (DAD), using a chiral AD-H column (5 μm, 250 × 4.6 mm, Daicel, Tokyo, Japan). Mouse macrophage cells and three human cancer cell lines (HCT-116, HepG2 and BGC-823) were obtained from the Cell Bank of the Chinese Academy of Sciences (Shanghai, China). A microplate reader (Thermo Fisher Scientific, Inc., Waltham, MA, USA) was used for the cytotoxicity assays.

### 3.2. Plant Material

*P. ensiformis* was collected in July 2013, from Wugui Mountain in Zhongshan City, Guangdong Province, China, and identified by Associate Professor Xiao-Zhong Chen (Heilongjiang University of Chinese Medicine). A voucher specimen (ID-g-20140726) has been deposited in the herbarium, Faculty of Jiamusi, Heilongjiang University of Chinese Medicine.

### 3.3. Extraction and Isolation

The whole plant of *P*. *ensiformis* (10 kg) was powdered and extracted with 95% EtOH (30 L × 3, 1 h each) under reflux conditions and the solvent was then evaporated under vacuum to yield an EtOH extract (1000 g). The EtOH extract was chromatographed on a polyamide resin chromatography column eluted with different EtOH/H_2_O (0%, 30%, 50%, 95%, *v*/*v*) isocratic solvent mixtures to afford four fractions (A–D). Fraction B (100 g) was subjected to MCI column chromatography eluting with an EtOH/H_2_O (0–95%, *v*/*v*) gradient solvent system to give four subfractions (Fr. B1-B4) on the basis of TLC detection. Fr. B3 (18 g) could be separated into 23 fractions by using MPLC column chromatography eluting with a MeOH/H_2_O (10–100%, *v*/*v*) gradient solvent system. Compounds **8** (5 mg), **9** (8 mg) and **10** (7 mg) were obtained from the tenth, tenth, and twelfth fraction, separately, by using prep-HPLC (MeOH/H_2_O 20%, 28, 28, *v*/*v*). Fr. C (300 g) was chromatographed on a polyamide resin column eluted with various EtOH/H_2_O (0, 30%, 50%, 95%, *v*/*v*) isocratic solvent systems to afford four subfractions (C1–C4). Furthermore, subfraction C3 (65 g) was subjected to MPLC column chromatography eluting with a MeOH/H_2_O (10–100%, *v*/*v*) gradient solvent system to yield 25 fractions. By using prep-HPLC, compounds **3** (12 mg) and **4** (31 mg) were isolated from subfraction C3(8); compounds **5** (7 mg)**, 6** (10 mg) and **7** (20 mg) were obtained from subfraction C3(10). Subfraction C3(5) was subjected to prep-HPLC to yield compound **1** (5 mg). Sequent chiral separation of compound **1** was performed to get the pair of enantiomers (+)-**1** (2 mg) and (−)-**1** (2 mg).

*3′,4-Dihydroy-3,4′-dimethoxy-dihydrochalcone* (**1**): Yellow powder; IR *ν*_max_ 3376, 1660, 1592, 1273, 1036, 843, 636 cm^−1^; ^1^H-NMR and ^13^C-NMR data see Table 1; HR-ESI-MS *m*/*z* 319.1176 [M − H]^+^ (calcd. for C_17_H_17_O_6_ *m*/*z* 318.1103); (+)-(β*R*)-**1**, ([α]${}_{D}^{25}$ + 15.0 (*c* 0.2, CH_3_OH), Rh_2_(OCOCF3)_4_-induced CD (MeOH) nm (Δε) 340 (+0.30); (−)-(β*S*)-**1**, ([α]${}_{D}^{25}$ − 15.1 (*c* 0.2, CH_3_OH), Rh_2_(OCOCF3)_4_-induced CD (MeOH) nm (Δε) 340 (−0.60). ^1^H-NMR (600 MHz, CD_3_OD): *δ* 7.56 (1H, d, *J* = 2.0 Hz, H-2), 6.80 (1H, d, *J* = 8.5 Hz, H-5), 7.62 (1H, dd, *J* = 8.5, 2.0 Hz, H-6), 4.26 (1H, dd, *J* = 8.5, 11.0 Hz, H-α1), 3.71 (1H, dd, *J* = 8.5, 5.5 Hz, H-α2), 4.76 (1H, dd, *J* = 8.5, 5.0 Hz, H-β), 6.89 (1H, d, *J* = 2.0 Hz, H-2′), 6.73 (1H, d, *J* = 8.5 Hz, H-5′), 6.76 (1H, dd, *J* = 8.5, 2.0 Hz, H-6′), 3.86 (3H, s, 3-OCH_3_), 3.82 (3H, s, 4′-OCH_3_); ^13^C-NMR (150 MHz, CD_3_OD): *δ* 130.3 (C-1), 112.5 (C-2), 149.0 (C-3), 153.3 (C-4), 115.7 (C-5), 125.2 (C-6), 199.6 (C=O), 65.5 (C-α), 56.3 (C-β), 129.9 (C-1′), 112.7 (C-2′), 149.3 (C-3′), 147.0 (C-4′), 116.6 (C-5′), 112.2 (C-6′), 56.3 (3-OCH_3_), 56.4 (4′-OCH_3_).

### 3.4. Cytotoxic Activity Assays

Three human cancer cell lines, HCT-116, HepG2 and BGC-823, were used in the cytotoxicity bioassay. All the cells were cultured in RPMI-1640 or DMEM medium (Hyclone, Logan, UT, USA), supplemented with 10% fetal bovine serum (Hyclone) in 5% CO_2_ at 37 °C. The cytotoxicity assay was performed according to the MTT (3-(4,5-dimethylthiazol-2-yl)-2,5-diphenyltetrazolium bromide) method in 96-well microplates [18]. Briefly, 100 μL adherent cells were seeded into each well of 96-well cell culture plates and allowed to adhere for 12 h before drug addition, while suspended cells were seeded just before drug addition with initial density of 1 × 10^5^ cells/mL. Each tumor cell line was exposed to the test compound at concentrations of 0.064, 0.32, 1.6, 8, and 40 μM in triplicates for 48 h, with taxol (Sigma, Ronkonkoma, NY, USA) as a positive control. After each compound treatment, cell viability was detected and cell growth curve was graphed. Half maximal inhibitory concentration (IC_50_) value was calculated by Reed and Muench’s method [19].

### 3.5. Inhibitory Assay of NO Production

C57BL6/J mouse macrophages were maintained in RPMI1640 medium at 37 °C in 5% CO_2_. The cells were placed in 48-well plates and preincubated for 24 h, treated with tested triterpene glycosides dissolved in DMSO at various final concentrations (0.2, 1.0, 5.0, 25.0, 125.0 μM) in triplicate for 1 h, and continuously incubated with LPS (1 μg/mL) for 24 h. Dexamethasone (10^−6^ M) was used as the positive control. From each well, the supernatants (100 μL) were mixed with an equal amount of Griess reagent at room temperature for 20 min. The concentration of NO_2_^−^ was measured for the amount of NO by a microplate reader at 570 nm, using sodium nitrite as the standard to calculate the concentration of the nitrite [20,21].

### 3.6. Cytotoxicity Assays

Cell viabilities were measured using the MTT assay. Briefly, mouse macrophages cells were seeded in 96-well plates at concentrations of 1 × 10^5^ cells per well. After incubation for 2 h, the cells were incubated with compounds for 24 h and then washed with PBS three times. Following the washing step, 200 μL of RPMI 1640 medium containing 0.5 mg/mL MTT were added to each well, the cells were then incubated at 37 °C for another 4 h. Finally, the culture medium was removed, and the formazan crystal was dissolved by adding 150 μL of DMSO. Absorbances at 570 nm were measured using a microplate reader.

### 3.7. In Vivo Anti-Inflammatory Assays

The 95% EtOH and polyamide resin at 30% ethanol extracts were tested for their in vivo anti-inflammatory activity on ear edema induced by croton oil in mice as per the established method [22]. Dexamethasone was used as the positive control.

## 4. Conclusions

Phytochemical investigation on *P. ensiformis* led to the isolation of a pair of new dihydrochalcone enantiomers (+)-**1** and (−)-**1**, together with eight known compounds **3**–**10**. Compounds (+)-**1** and (−)-**1** are dihydrochalcones, **3** and **4** are hydrochalcones, **5**–**7** are flavonoids, , and **8**–**10** are diterpenoids. Results of an in vitro study of the biological activity showed that compounds (+)-**1**, (−)-**1**, **8**, **9** and **10** exhibited inhibitory effects on macrophage activation, suggesting that these compounds have potential as anti-inflammatory disease agents. Compound **10** showed moderate cytotoxic activity against HCT-116, HepG-2 and BGC-823 cell lines.

## Figures and Tables

**Table 1:** ^1^H-NMR and ^13^C-NMR data of **1** (600/150 MHz, Methanol-*d*_4_).

Position	*δ*_C_	*δ*_H_ (*J* in Hz)	HMBC
1	130.3		
2	112.5	7.56, d, 2.0	C-1, C-3, C-4, C-6, C=O
3	149.0		C-1, C-2, C-4, C-5
4	153.3		
5	115.7	6.80, d, 8.5	C-1, C-3, C-4, C-6
6	125.2	7.62, dd, 8.5, 2.0	C-1, C-2, C-4, C-5
C=O	199.6		
α	65.5	4.26, dd, 11.0, 8.5, 3.71, dd, 11.0, 5.5	C-1, C-1′, C=O, C-β
β	56.3	4.76, dd, 8.5, 5.0	C-1′,C-2′, C-6′, C=O, C-α
1′	129.9		C-α, C-β, C-2′, C-3′, C-5′, C-6′
2′	112.7	6.89, d, 2.0	C-β, C-1′,C-3′, C-4′, C-6′
3′	149.3		
4′	147.0		
5′	116.6	6.73, d, 8.5	C-1′, C-3′, C-4′, C-6′
6′	122.2	6.76, dd, 8.5, 2.0	C-β, C-1′,C-2′, C-4′, C-5′
3-OCH_3_	56.3	3.86, s	C-3
4′-OCH_3_	56.4	3.82, s	C-4′
			

**Table 2:** Effects of the plant extracts on ear edema induced by croton oil in mice (*n* = 3).

Extracts	Dose (mg/kg)	Edema Degree (mg)	Inhibitation Rate (%)
95% Ethanol extract	200.0	13.89 ± 3.25 *	38.6
polyamide resin at 30% ethanol extract	200.0	9.89 ± 1.33 **	56.3
Dexamethasone ^a^	1.0	6.61 ± 0.89 **	70.8
Control group	-	22.63 ± 3.16	-

^a^ Positive control; * *p* < 0.05 vs. control group; ** *p* < 0.01 vs. control group.

**Table 3:** Inhibitory effects of compounds against LPS-induced NO production in mouse macrophage cells (*n* = 3).

Compounds	IC_50_ (μM)	Compounds	IC_50_ (μM)
(+)-1	2.0 ± 0.21	7	>50
(−)-1	2.5 ± 0.82	8	8.0 ± 0.98
3	>50	9	9.5 ± 0.75
4	>50	10	5.6 ± 0.69
5	>50	dexamethasone *^a^*	0.025 ± 0.37
6	>50		

*^a^* Positive control.

## Supplementary Materials

Supplementary materials are available online.
