# Supplementary material for: Dihydrochalcones and Diterpenoids from Pteris ensiformis and Their Bioactivities
Source: Molecules. 2017 Aug 25;22(9):1413. doi: 10.3390/molecules22091413 (PMC6151822; doi:10.3390/molecules22091413)
Supplement: Supplementary file 1 [file molecules-22-01413-s001.pdf]

Dihydrochalcones and Diterpenoids from *Pteris ensiformis* and their Bioactivities

Supplementary Materials

List of Contents

Fig. S1 The IR Spectrum of **1**

Fig. S2 The ^{1}H NMR Spectrum of **1** in MeOH-d_4 (600 MHz)

Fig. S3 The ^{13}C NMR Spectrum of **1** in MeOH-d_4 (150MHz)

Fig. S4 The gHSQC Spectrum of **1** in MeOH-d_4 (600 MHz)

Fig. S5 The gHMBCSpectrum of **1** in MeOH-d_4 (600 MHz)

Fig. S6 The gCOSYSpectrum of **1** in MeOH-d_4 (600 MHz)

Fig. S7 HRESIMS spectrum of **1**

Fig. S8 The ^{1}H NMR Spectrum of (+)-**1** in MeOH-d_4 (600 MHz)

Fig. S9 The ^{1}H NMR Spectrum of (-)-**1** in MeOH-d_4 (600 MHz)

Fig. S10 The CD Spectra of (+)-**1** (blue) and (-)-**1** (black).

Fig. S11 $^{1}H-^{1}H$ COSY and HMBC correlations of **1**

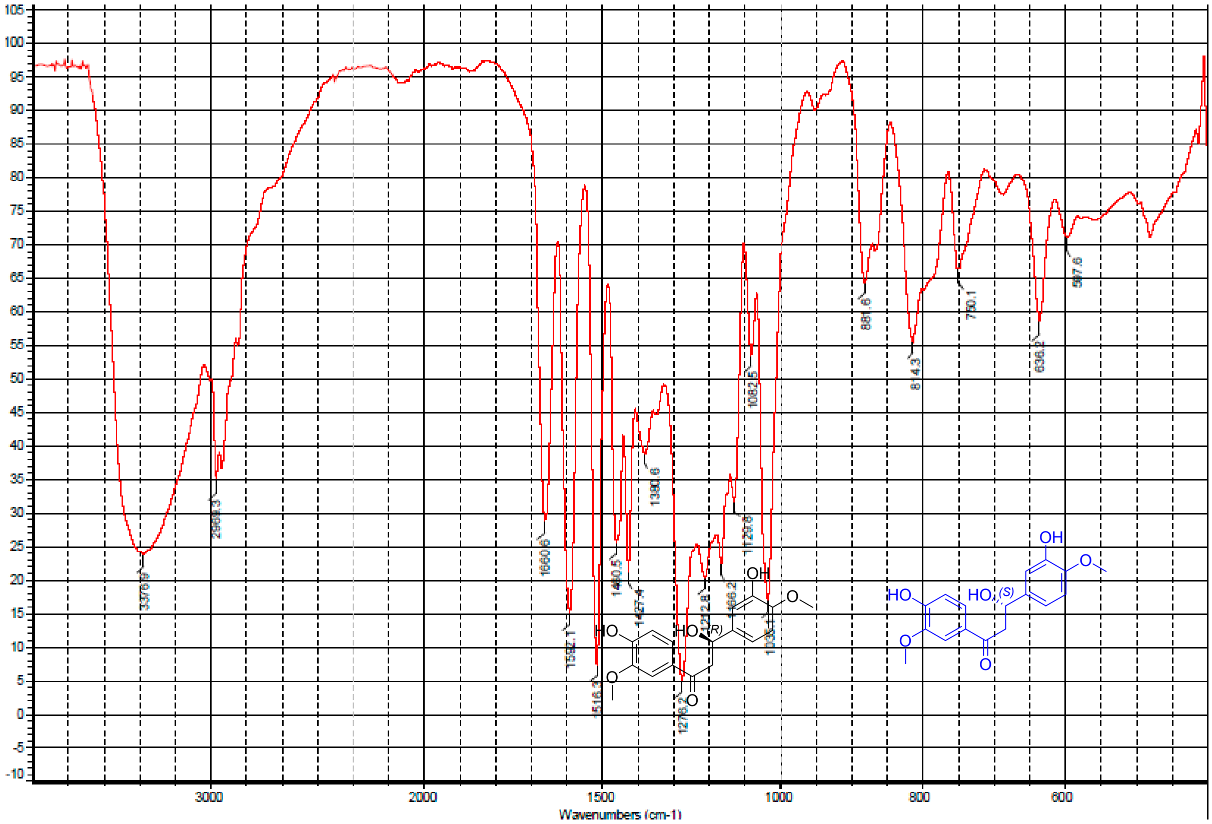

Fig. S1 IR spectrum of compound **1**

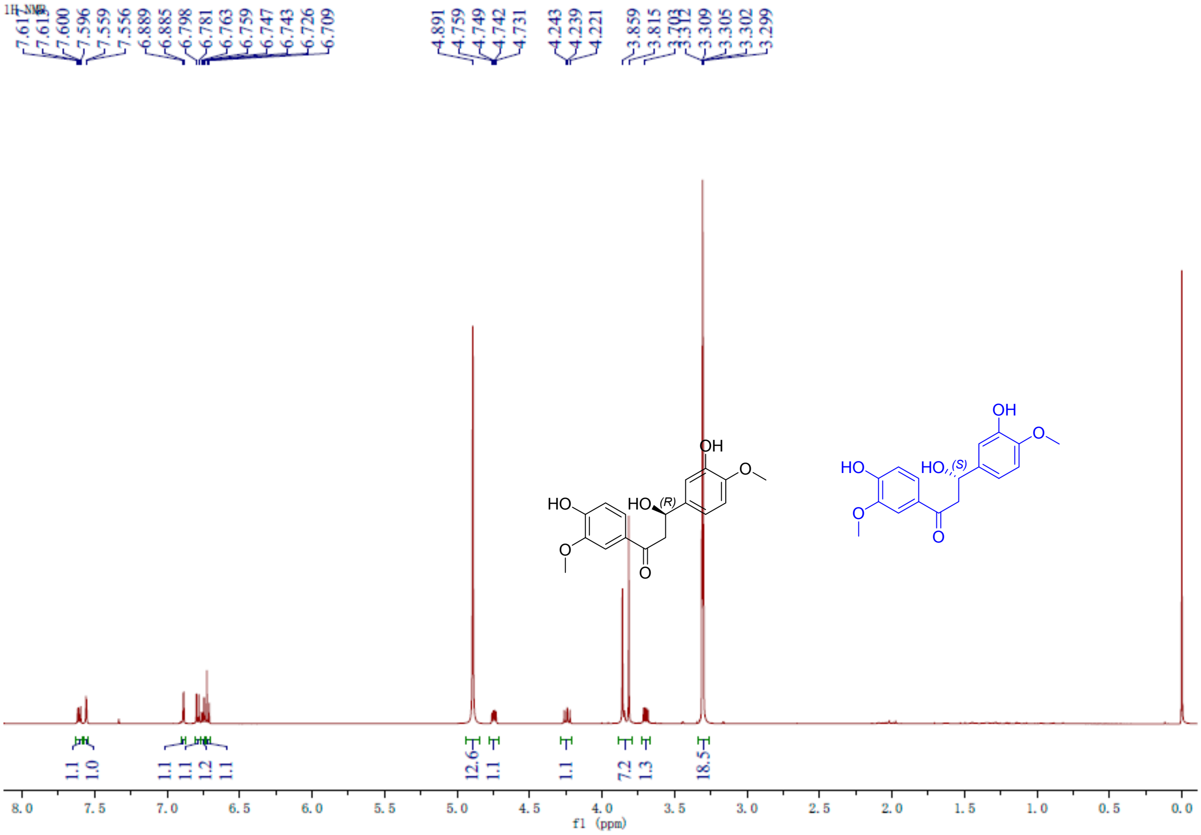

Fig. S2 ¹H-NMR spectrum of compound **1** in MeOH-d_4 (600 MHz)

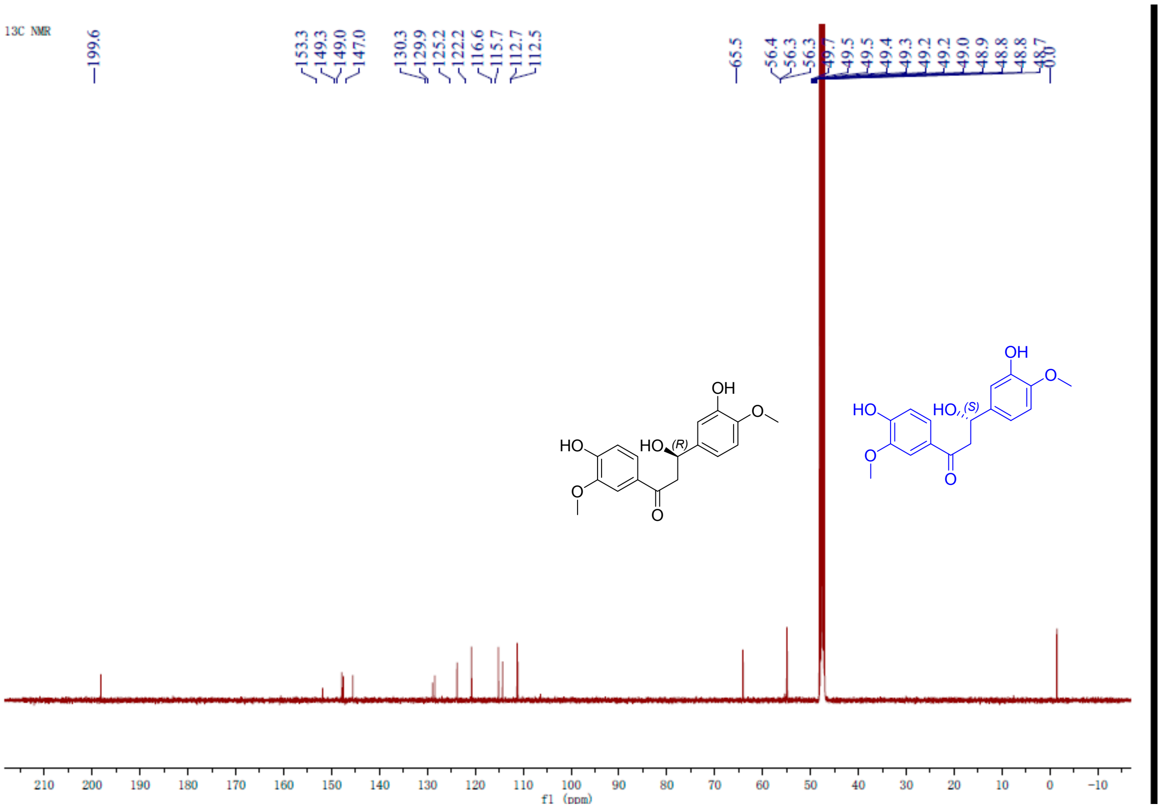

Fig. S3 ^{13}C-NMR spectrum of compound **1** in MeOH-d_4 (150 MHz)

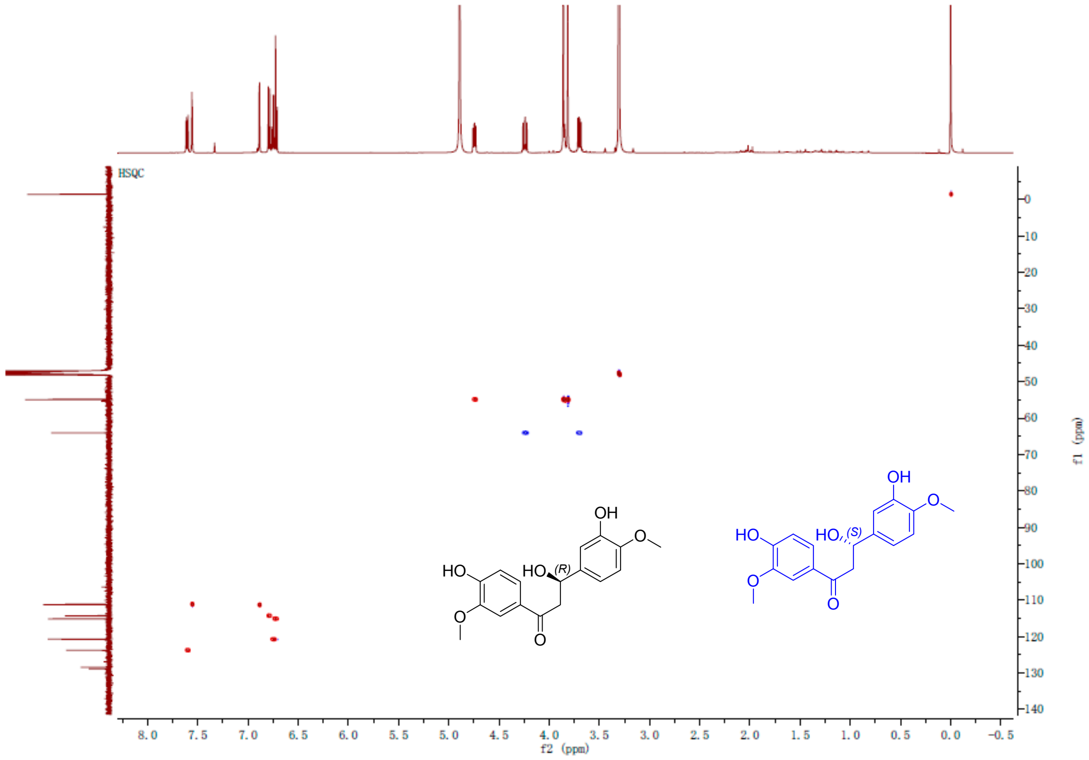

Fig. S4 HSQC spectrum of compound **1** in MeOH-d_4 (600 MHz)

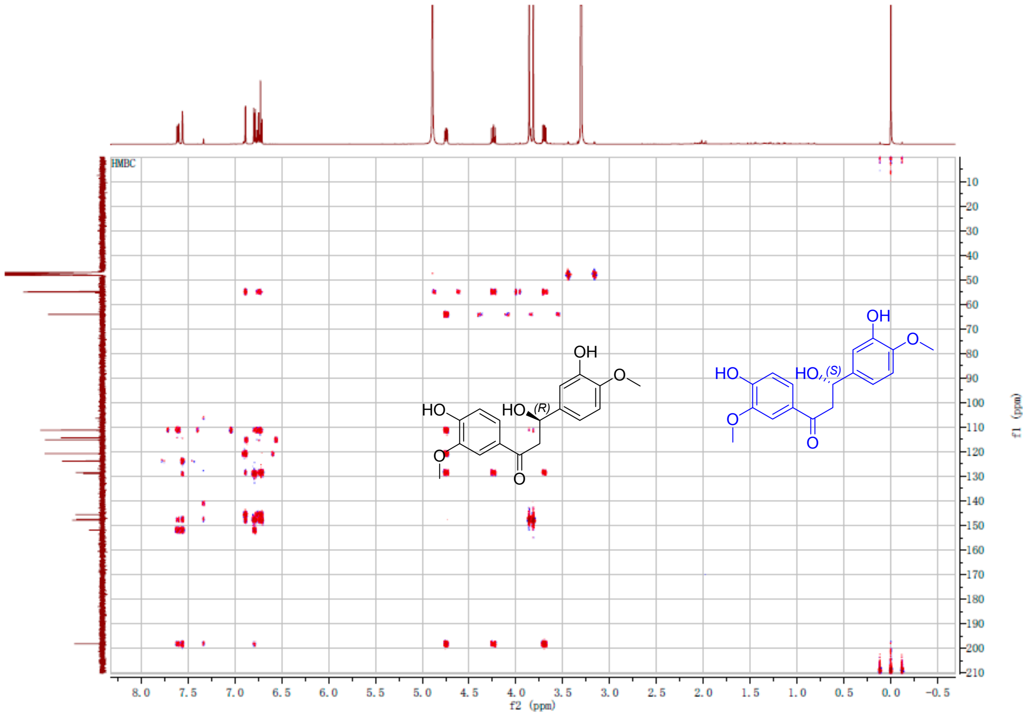

Fig. S5 HMBC spectrum of compound **1** in MeOH-d_4 (600 MHz)

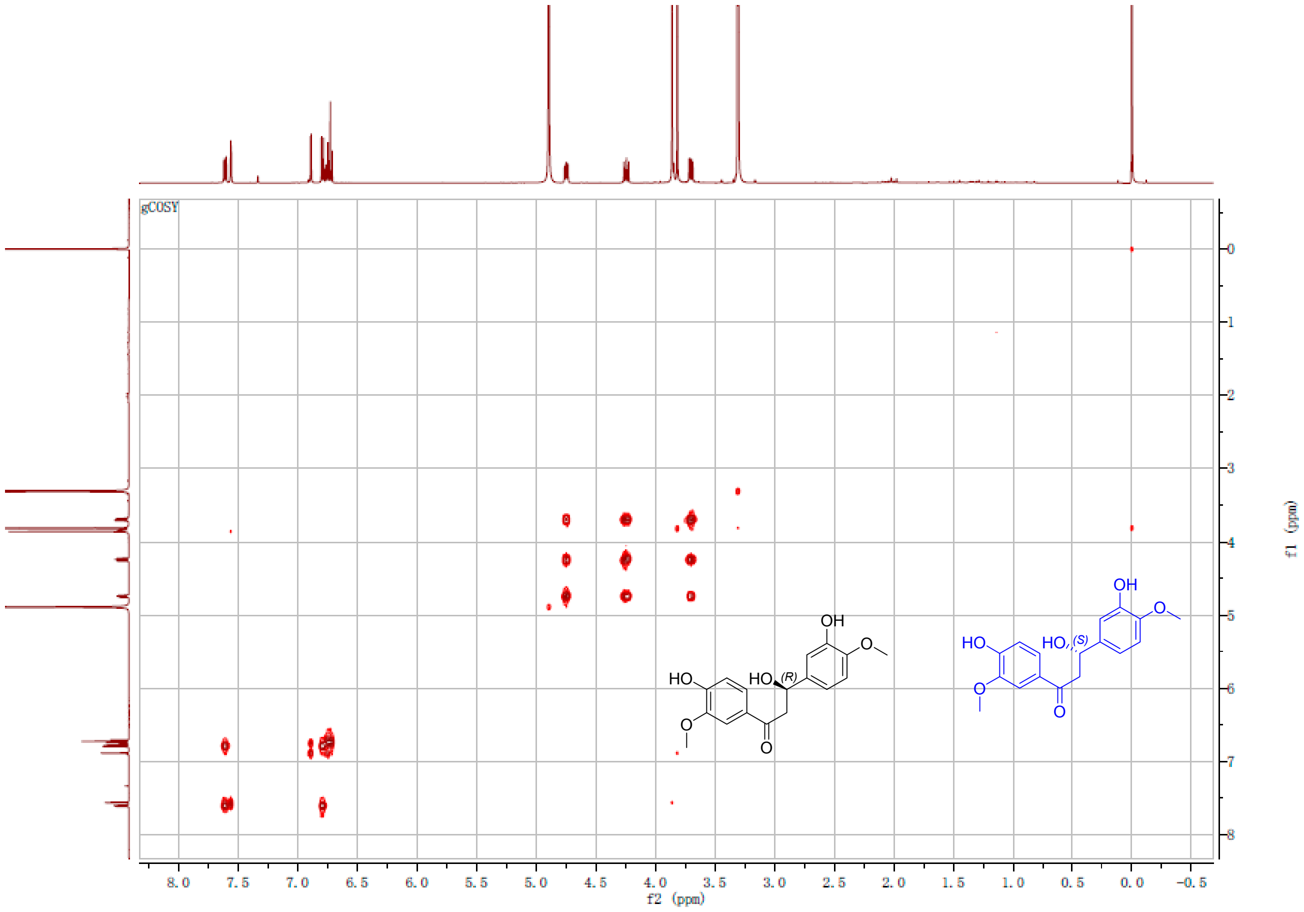

Fig. S6 ^1H-^1H COSY spectrum of compound **1** in MeOH-d_4 (600 MHz)

MS Formula Results: + Scan (4.752 min) Sub (2015101201.d)

m/z	Ion	Formula	Abundance
319.1177	(M+H)+	C17 H19 O6	1124030

Best	Formula (M)	Ion Formula	Score	Cross Sco	Mass	Calc Mass	Calc m/z	Diff (ppm)	Abs Diff (ppm)	Mass Match	Abund Match	Spacing Match	DBE
☑	C17 H18 O6	C17 H19 O6	99.99		318.1104	318.1103	319.1176	-0.27	0.27	100	99.97	99.99	9
☐	C18 H14 N4 O2	C18 H15 N4 O2	99.58		318.1104	318.1117	319.119	3.92	3.92	99.52	99.36	99.98	14

Fig. S7 HRESIMS spectrum of compound **1**

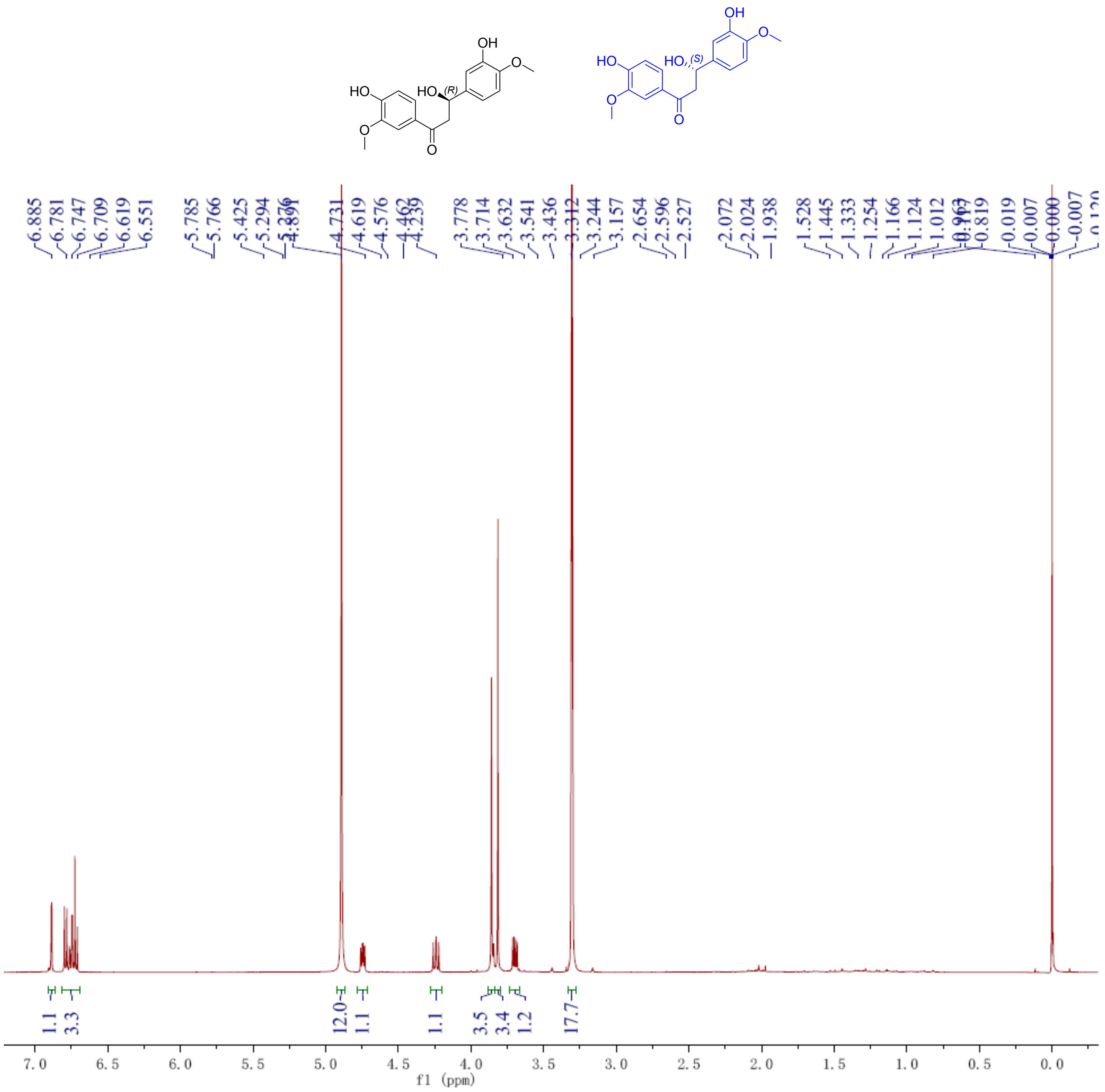

Fig. S8 The 1H NMR Spectrum of (+)-**1** in MeOH-d_4 (600 MHz)

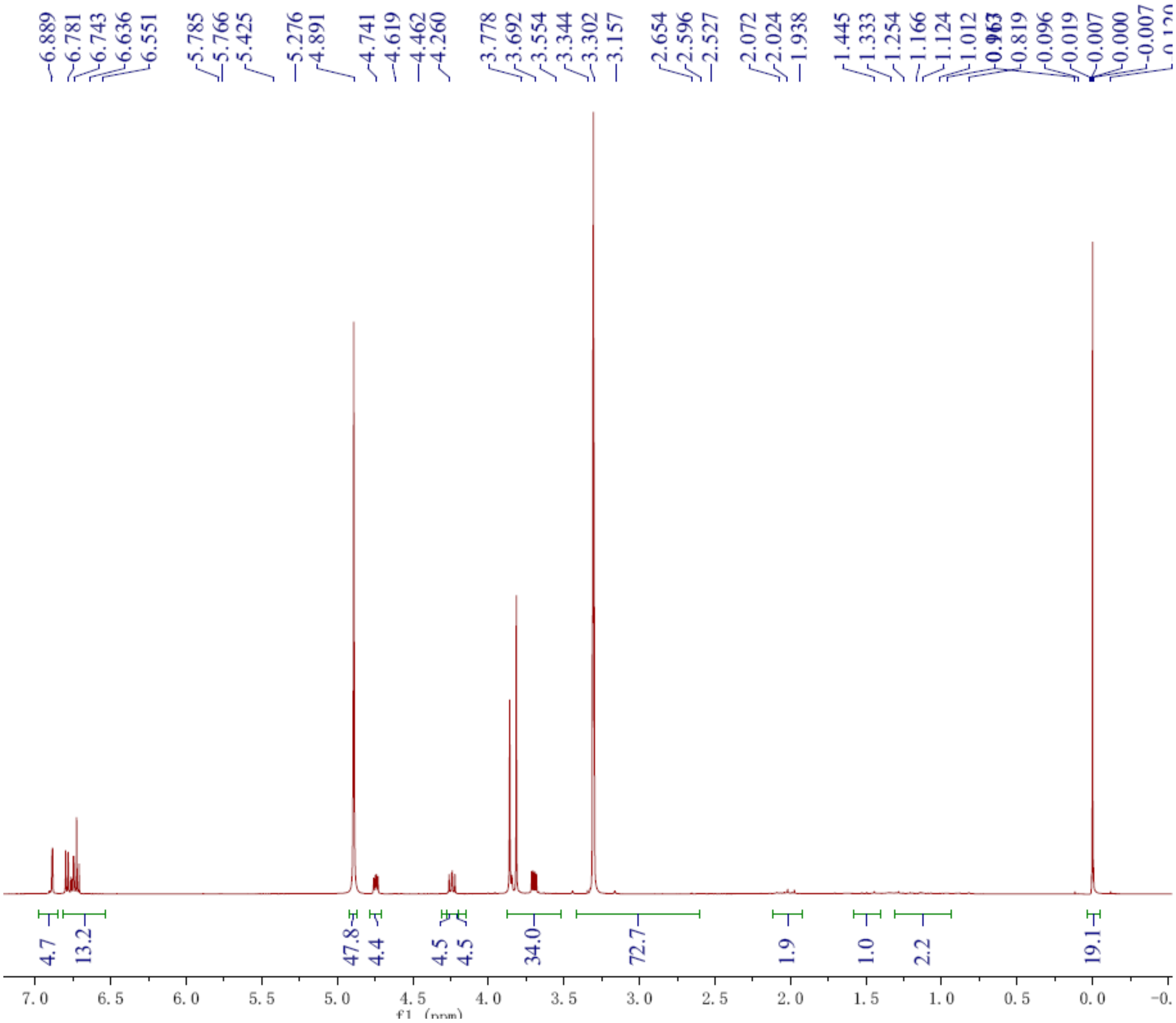

Fig. S9 The 1H NMR Spectrum of (-)-**1** in MeOH-d_4 (600 MHz)

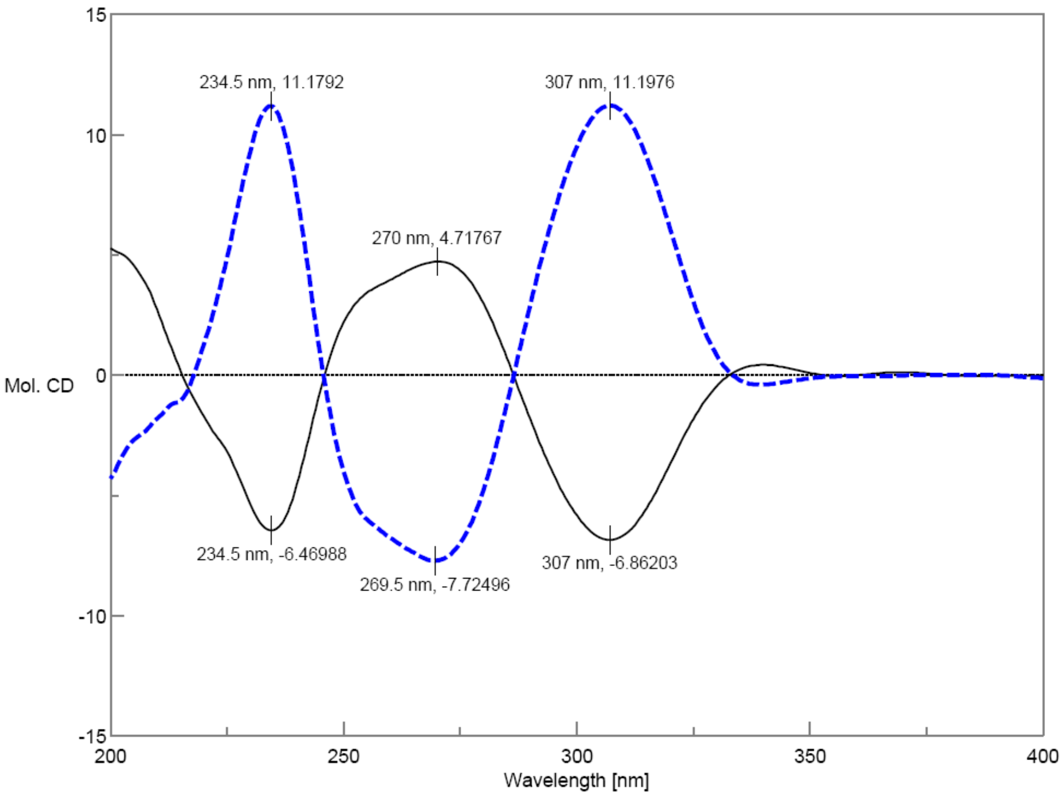

Fig. S10 The CD Spectra of (+)-**1** (blue) and (-)-**1** (black).

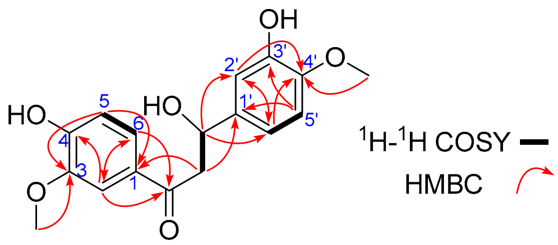

Fig. S11 ^{1}H−^{1}H COSY and HMBC correlations of **1**
